# Phylogeography of the Golden Jackal (*Canis aureus*) in India

**DOI:** 10.1371/journal.pone.0138497

**Published:** 2015-09-28

**Authors:** Bibek Yumnam, Tripti Negi, Jesús E. Maldonado, Robert C. Fleischer, Yadvendradev V. Jhala

**Affiliations:** 1 Wildlife Institute of India, Chandrabani, Dehradun 248001, India; 2 Smithsonian Conservation Biology Institute, National Zoological Park, 3001 Connecticut Avenue, Washington, D.C. 20008, United States of America; 3 Department of Vertebrate Zoology, National Museum of Natural History, Smithsonian Institution, Washington, D.C. 20013, United States of America; BiK-F Biodiversity and Climate Research Center, GERMANY

## Abstract

The golden jackal (*Canis aureus*) is one of the most common and widely distributed carnivores in India but phylogeographic studies on the species have been limited across its range. Recent studies have observed absence of mitochondrial (mt) DNA diversity in European populations while some North African populations of golden jackal were found to carry gray wolf (*Canis lupus lupaster*) mtDNA lineages. In the present study, we sequenced 440 basepairs (bp) of control region (CR) and 412 bp of cytochrome *b* (cyt *b*) gene of mtDNA from 62 golden jackals sampled from India (n = 55), Israel (n = 2) and Bulgaria (n = 5), to obtain a total of eighteen haplotypes, comprising sixteen from India and one each from Israel and Bulgaria. Except for three previously described haplotypes represented by one cyt *b* and one CR haplotype both from India, and one CR haplotype from Bulgaria, all haplotypes identified in this study are new. Genetic diversity was high in golden jackals compared to that reported for other canids in India. Unlike the paraphyletic status of African conspecifics with the gray wolf, the Indian (and other Eurasian) golden jackal clustered in a distinct but shallow monophyletic clade, displaying no evidence of admixture with sympatric and related gray wolf and domestic dog clades in the region. Phylogeographic analyses indicated no clear pattern of genetic structuring of the golden jackal haplotypes and the median joining network revealed a star-shaped polytomy indicative of recent expansion of the species from India. Indian haplotypes were observed to be interior and thus ancestral compared to haplotypes from Europe and Israel, which were peripheral and hence more derived. Molecular tests for demographic expansion confirmed a recent event of expansion of golden jackals in the Indian subcontinent, which can be traced back ~ 37,000 years ago during the late Pleistocene. Our results suggest that golden jackals have had a potentially longer evolutionary history in India than in other parts of the world, although further sampling from Africa, the Middle East and south-east Asia is needed to test this hypothesis.

## Introduction

The golden jackal (*Canis aureus*, Linneaus 1758) is a medium-sized canid with a wide range of distribution, occurring in northern and eastern Africa, southeastern Europe, the Middle East and extending eastwards into central, southern and southeast Asia as far south as Sri Lanka and Thailand [1]. It is included in CITES Appendix III which permits limited trade of pelts. In India, the golden jackal is listed in Schedule III of the Wildlife Protection Act (1972, [2]), which though offers reduced protection compared to endangered species, but still ensures a complete ban on hunting. The species is not at risk of extinction, and there is evidence of recent expansion assisted by recent wolf extermination and partial legal protection as observed in south-eastern Europe [3, 4].

Unlike the closely related and much rarer sympatric species, the gray wolf (*Canis lupus*), the golden jackal is fairly common throughout its range. Due to their tolerance of dry habitats and omnivorous diet, they can live in a wide variety of habitats. Golden jackals are opportunistic and often venture into human habitations at night to feed at garbage dumps, or scavenge on livestock carcasses. In India, the golden jackal is found in most protected areas, semi-urban and rural landscapes of the country, except in the high elevation regions of the Himalaya. Certain pastoral areas in western and northern India, which have abundant livestock also support high jackal densities. This is mainly because many cattle carcasses are available for jackals and other carnivores to scavenge upon, as prevailing socio-religious beliefs among the people in this region taboo the consumption of beef [1]. Based on intensive observations on breeding pack units and radio-collared individuals, jackal densities in the semi-arid Velavadar National Park were estimated between one and two jackals per km² [5]. On the African continent, in the Serengeti National Park, densities can range to as high as four adults per km² [6].

Despite their wide range of distribution, limited population genetic studies on the golden jackal have been carried out [7, 8, 9]. Preliminary studies were restricted to resolving phylogenetic relationships among wolf-like canids [10] or investigating cryptic speciation of African golden jackals [11, 12, 13] and detecting hybridization events between gray wolves and golden jackals in Bulgaria [14]. Recent studies in Europe [7, 9] have observed mtDNA genetic diversity in golden jackal to be surprisingly low despite its wide range of distribution. Zachos et al. [7] and Fabbri et al. [9] respectively sampled 121 and 120 golden jackal individuals from Serbia, Croatia, Bulgaria and Italy, but detected only a single mtDNA haplotype across the entire area. These findings suggest successive waves of demographic contraction and rapid expansion from a limited set of founders near the north-western limit of their range in Europe. Fabbri et al. [9] also detected low diversity at 15 microsatellite loci (average heterozygosity ~ 0.3 to 0.5). Comparatively higher variation at 14 microsatellite loci was observed in 88 golden jackals sampled from Israel (average heterozygosity ~ 0.4 to 0.7) and with no evidence of genetic bottleneck, despite being nearly exterminated from the area more than four decades ago [8]. Recently, some north African canids within the morphologically delineated golden jackal group were detected to carry mtDNA haplotypes falling within the gray wolf clade [11, 12, 13], thus leading to it being re-classified as a subspecies of the gray wolf (*Canis lupus lupaster*). Although interpreted as a taxonomic misidentification, this entity could have resulted from the introgression of gray wolf mtDNA into golden jackal populations, as observed from the ‘intermediate’ or hybrid jackal-wolf morphotypes observed at a Senegalese study site by Gaubert et al. [12]. However, a recent comprehensive study of African and Eurasian golden jackals, based on mitochondrial and nuclear genome sequences, has found strong support to merit it’s recognition as a genetically distinct canid species that diverged ~1 million years ago from related Eurasian golden jackals and gray wolves [13]. Limited hybridization between golden jackal and gray wolf has also been observed in Europe, in the Balkans [14] and possibly in the Caucasus as well [15]. Hitherto regarded as a species of low conservation significance and therefore meriting limited genetic investigations, these recent studies underscore the need for genetic studies to understand the phylogeography and diversity of golden jackals throughout their extant range.

In this paper, we present to our knowledge a first assessment on the genetic variability of the golden jackal populations in India. The Indian golden jackal populations are important for investigating phylogeography in the species as they encompass a major central portion of their global distribution. This study used mtDNA sequences to explore the following themes with regard to golden jackal systematics and phylogeography in India—(i) genetic diversity among golden jackal populations in India, (ii) signatures of population change, and (iii) relationships to wolves and other wolf-like canids in the region and across their range.

## Materials and Methods

### Ethics Statement

Samples were collected from road-killed individuals and from golden jackals that were captured for a radio-telemetry study in Gujarat. Golden jackals were captured using a rubber padded "soft catch" foot hold trap and subsequently anesthetized using a gas powered dart delivery system with a combination of 8mg/kg ketamine and 2mg/kg xylazine [16]. Blood samples were drawn by a qualified veterinarian and jackals were cared for till total recovery from anesthesia, released at the capture site and subsequently monitored through radio-signals to ensure their well-being. Capture permits were obtained from the Chief Wildlife Warden as per the Wildlife (Protection) Act 1972. *Canis aureus* is listed in schedule III of this Act, and as species of least concern by the IUCN red list. This research project was conceived and radio collaring of jackals was done prior to the formation of an animal ethics committee at the Wildlife Institute of India. The research was approved by the Training and Academic Council (TRAC) of the Wildlife Institute of India which is composed of experienced wildlife biologists, academicians, wildlife managers, the dean and director of the Wildlife Institute. The TRAC approves research proposals based on their scientific merit and value for conservation. Use of soft catch leg-hold traps, capture techniques and anesthesia for canids were approved by TRAC and the Chief Wildlife Warden. Smithsonian Institution scientists were not involved in the field ecology study and were subsequently involved in study design and training of two student co-authors in the genetics portion of the study. Furthermore, none of the samples were processed in the genetics lab at the Smithsonian Institution. All laboratory analysis was done at the Conservation Genetics laboratory of the Wildlife Institute of India. Samples from Bulgaria and Israel were provided by Ivan Nikolov (Technical University Munich, Germany) and Dr. Eli Geffen (Tel Aviv University, Israel) respectively.

### Sampling and Laboratory work

Tissue (n = 31) and hair samples (*n* = 7) were obtained opportunistically from road-killed individuals of golden jackal from across northern, western and peninsular India between the years 1994 to 2012 (S1 Table and S1 Fig). Blood samples (*n* = 11) were obtained from golden jackals that were captured for a telemetry study in Gujarat in western India. Two samples from Israel and five samples from Bulgaria were also obtained for analyses. Samples were collected in 15 ml polypropylene screw cap tubes containing 70% ethanol in field and stored at -20°C upon arrival at the lab. Total DNA was extracted using the DNeasy extraction kit (QIAGEN Ag., Germany). We amplified approximately 440 bp region of the mtDNA CR locus using the primer pairs ThrL15926 and DL-H16340 [17]; and a 412 bp fragment of the mtDNA cyt *b* gene using a canid specific light primer (Canid L1, [18]) and universal heavy primer (H15149, [19]). Polymerase chain reactions (PCRs) were carried out in a volume of 25 μl including 0.5 units of AmpliTaq Gold (Applied Biosystems Inc., USA), 1X PCR buffer, 2.0 mM of MgCl2, 0.2 mM of each deoxynucleoside triphosphate, 0.5 μM of each primer, and 100–200 ng of template DNA. The thermocycling parameters were—initial denaturation at 95°C for 15 minutes, followed by 35 cycles at 94°C for 30 seconds, 55°C for 30 seconds and 72°C for one minute, and a final elongation step at 72°C for ten minutes. PCR products were cleaned using ExoSAP (GE Healthcare, USA) and sequenced in both directions using the BigDye Terminator v3.1 Cycle Sequencing Kit (Applied Biosystems Inc., USA) according to the manufacturer’s instructions. DNA sequences were resolved on the ABI Prism 3130 Genetic Analyzer (Applied Biosystems Inc., USA). All sequences obtained from this study were aligned by eye in Sequencher 4.6 (Gene Codes Corporation Inc., USA), and deposited in GenBank under the accession numbers (KT343779 –KT343803, see S1 and S2 Tables).

### Data Analyses

To understand evolutionary relationships of golden jackals with other *Canis* sp., we performed a Bayesian Markov Chain Monte Carlo (MCMC) sampling scheme in MrBayes v3.2.2 [20] by using the concatenated fragment of the mtDNA cyt *b* (332 bp) and CR (277bp) sequences of golden jackals generated in this study, and including corresponding sequences of other wolf-like canids available in GenBank. Maned wolf (*Chrysocyon brachyurus*) sequence was used as outgroup to root the tree. The run length consisted of a total of 2 million MCMC replicates, of which the first 0.5 million runs comprised the burn-in, and chain convergence was assessed from the average standard deviation in split frequencies (<0.01). Gaps were treated as missing data and not used in analysis. Trees were analyzed using the Hasegawa-Kishino-Yano (HKY, [21]), general time reversible (GTR, [22]), Felsenstein 1981 (F81, [23]) and mixed models of molecular evolution implemented in Mr Bayes. We analyzed the outputs of MrBayes runs using Bayes Factors [24] to obtain estimates of probabilities for the best model of molecular substitution for our data (S3 Table). The GTR substitution model with a gamma distributed rate variation and having a proportion of invariable sites (GTR + invgamma) was found to be the most likely model (Model Probability = 0.981) compared to all other models tested (Model Probability < 0.02) in this study. We used MEGA 6 [25] to estimate mtDNA cyt *b* and CR genetic distances between species, by applying the Tamura-Nei model [26] and a Gamma distribution parameter of 0.3 as in Sharma et al. [27]. All gaps were treated as missing data and removed from analysis using the conservative *Complete-Deletion* option in MEGA. Standard error was estimated using 1,000 bootstrap replicates. To examine potential regional genetic structure, a median joining network tree [28] of mtDNA haplotypes was constructed using the program NETWORK 4.613 (http://www.fluxus-engineering.com, Accessed 20 June 2015). Network calculations were carried out by assigning equal weights to all variable sites and with default values for the epsilon parameter (epsilon = 0) in order to minimize alternative median networks. Gaps were treated as missing data and nucleotide alignment blocks containing indels were removed before analysis.

We used Arlequin v3.1 [29] to calculate the number of polymorphic sites (*S*), nucleotide (*Π*) and haplotype diversity (*h*). Nucleotide diversity is the average number of nucleotide differences per site and *h* is the probability that any two randomly sampled haplotypes are different [30]. Signatures of population expansion, equilibrium or decline in golden jackals were inferred from Tajima’s *D* [31] and Fu’s *F*_S_ [32] statistics, and mismatch distribution tests [33] were computed in DnaSP v.5.10 [34] and Arlequin v3.1 [29] using mtDNA CR sequences. Tajima’s test is based on average pairwise differences between sequences arising from the number of segregating polymorphic sites (*S*). Tajima’s *D* statistic is a measure of the observed variation seen in a population with respect to variation expected in a randomly evolving population at drift-mutation equilibrium. If the variation is selectively neutral and populations are at equilibrium, Tajima’s *D* will be indistinguishable from zero. On the other hand populations which experienced a contraction will have positive value of *D* (too few segregating sites/too many pairwise differences). *D* will be negative in populations which are undergoing demographic expansion or mutational selection (too many segregating sites/too few pairwise differences). The related Fu’s *F*_S_ statistic measures the discrepancy between nucleotide differences observed in the sample, compared to the distribution expected in a randomly evolving selectively neutral sample at population equilibrium. A significantly negative value of Fu’s *F*_*S*_ statistic indicates recent demographic expansion or departure from the null model hypothesis of selective neutrality and population equilibrium. The *P*-value is obtained from the proportion of random *F*_*S*_ statistics to the observed *F*_*S*_ value. Mismatch distribution tests detect patterns of variation of observed sequence differences to that expected in a randomly neutral population at drift-mutation-equilibrium. Populations that have experienced a recent expansion typically produce a right-skewed unimodal peak, whereas ragged and multimodal distributions are symptomatic of populations at demographic equilibrium [33]. To calculate the estimated time since expansion (*t*), the parameter *Tau* (*Τ*)τ was estimated from mismatch distributions of pairwise differences among golden jackal haplotypes, and the formula *Τ = 2μt* was rearranged to calculate *t*, where *μ* is the mutation rate of the control region in units of per base pair per generation. We used a mutation rate (*μ*) of 7.30 x 10^−5^ bp^-1^ yr^-1^ as previously applied to CR sequences in the gray (*Urocyon cinereoargentus*, [35]) and red fox (*Vulpes vulpes*, [36]). A generation time of one year was chosen based on the age of sexual maturity or first reproduction which is about eleven months in golden jackal [37].

## Results

**Fig 1:**
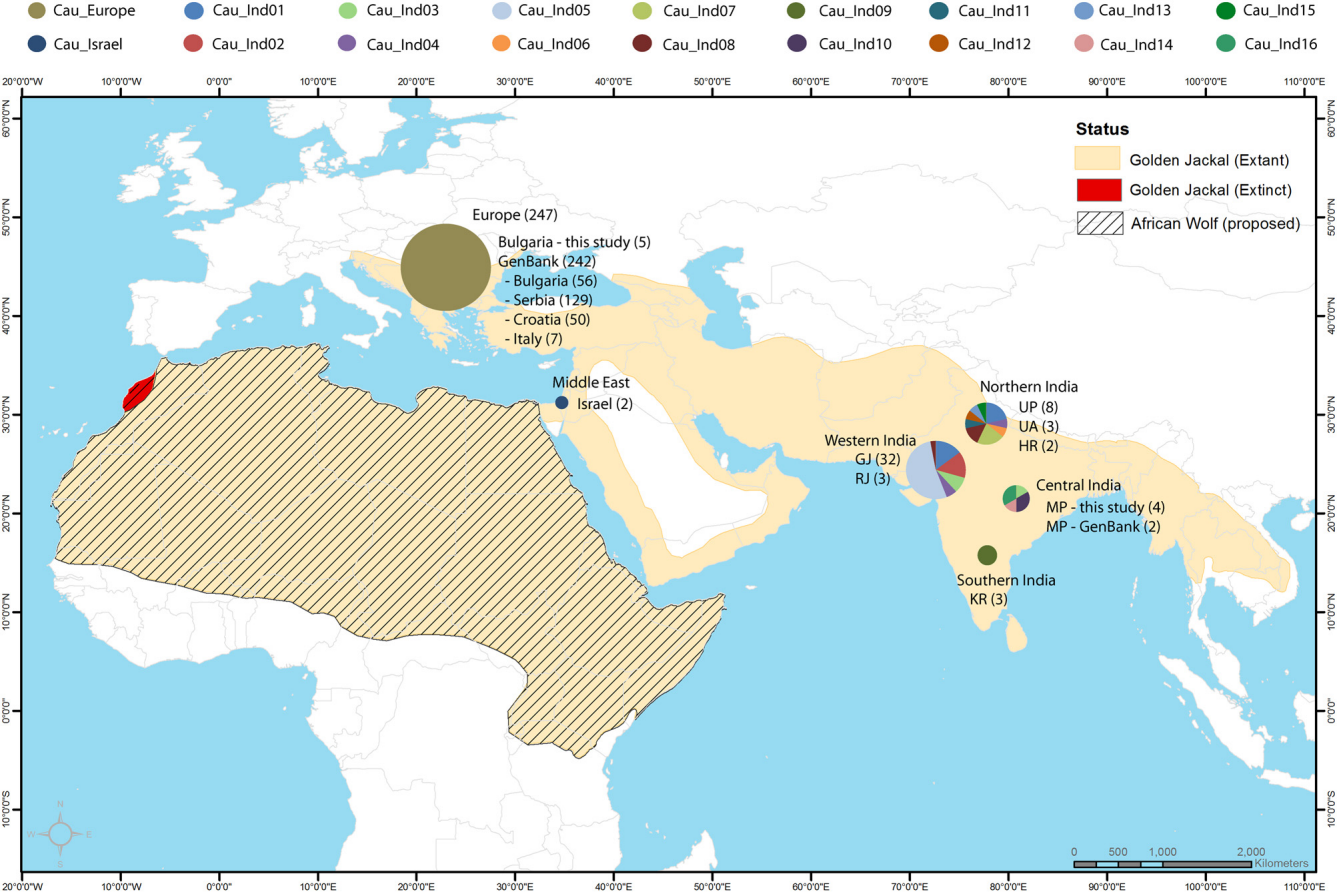
Map showing golden jackal range and geographic distribution of mtDNA control region haplotypes. Pie charts are proportional to haplotype frequency in each area. The distribution of the African wolf (or African golden jackal) is putative and based on recent studies in North Africa [11, 12, 13], which observed cryptic speciation or introgression of gray wolf mtDNA lineages within golden jackals and wolf-jackal hybrid phenotypes, implying that the African wolf (gray wolf mtDNA clade) likely co-occur in the same area as the golden jackal in Africa. The golden jackal distribution map was obtained as a shape-file from the IUCN species database (http://maps.iucnredlist.org/map.html?id=3744, Accessed 4 June 2014), while the base geographic layer was downloaded from the NaturalEarth database (www.naturalearthdata.com, Accessed 4 June 2014).

**Fig 2:**
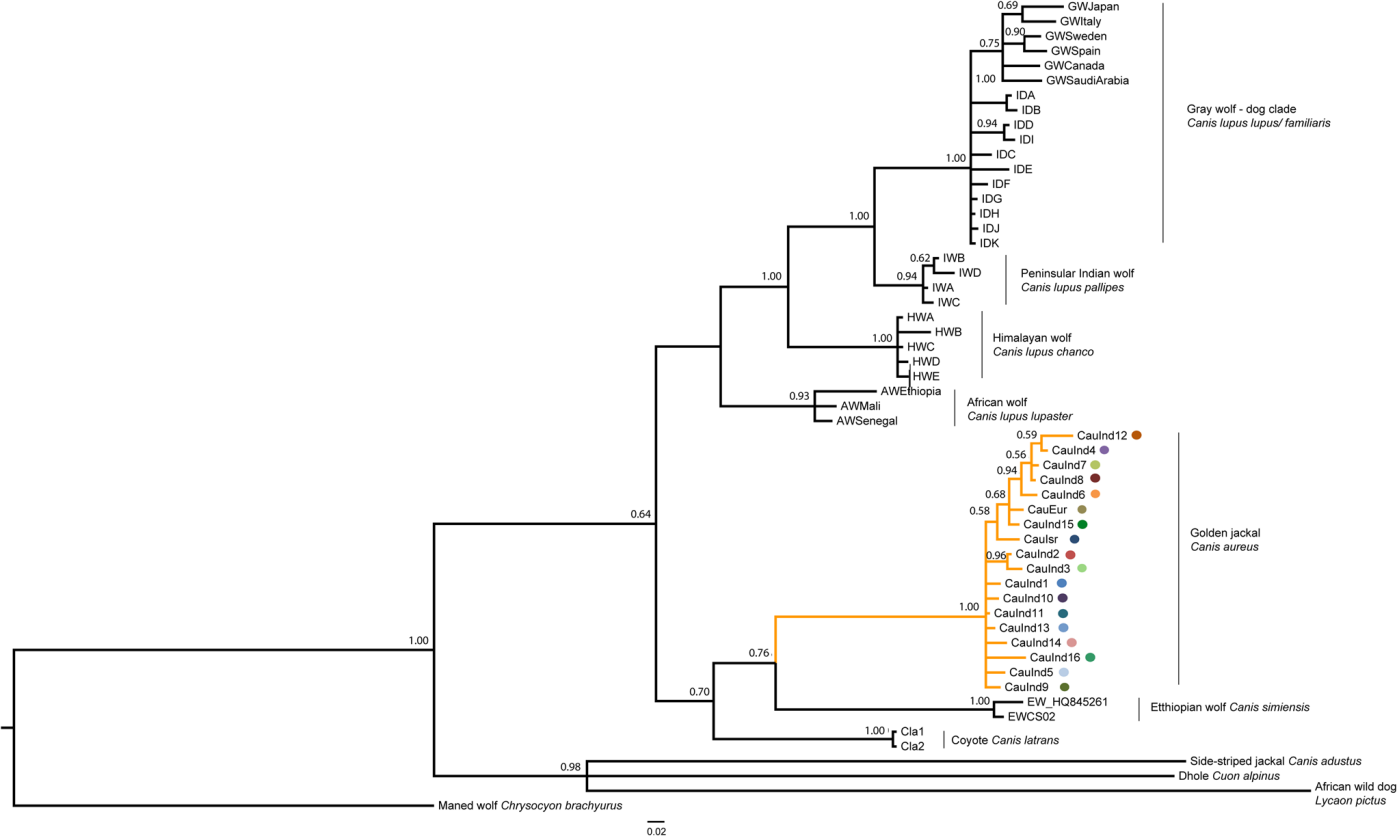
Bayesian phylogenetic relationships of the golden jackal clade based on concatenated cyt *b* (332 bp) and CR (277 bp) DNA sequences. Values at nodes correspond to posterior probabilities > 0.50. Scale bar represents 2% sequence divergence.

A total of eighteen CR haplotypes comprising sixteen from India, and one each from Israel and Bulgaria, were obtained from CR sequences of 58 golden jackals (56 from this study and two from GenBank) from India, the Middle East and Europe (Fig 1 and Table 1). In contrast, cyt *b* showed lower genetic diversity compared to CR sequences, with only nine haplotypes being obtained from a total of 40 cyt *b* sequences of golden jackal from India, Israel and Bulgaria. Seven cyt *b* haplotypes (six from this study and one from GenBank) were found in India (Table 2). CR and cyt *b* haplotypes from India were not present in Bulgaria or Israel, and vice-versa. All haplotypes sequenced in this study are new and not previously described in GenBank, except the Bulgarian CR haplotype which is identical to a previously published European haplotype sequence [7, 9, 38], and one cyt *b* and CR haplotype each from Central India [39]. All the Indian, Bulgarian and Israeli haplotypes formed a monophyletic clade within the golden jackal lineage which was strongly supported (posterior probability = 1.00) in the Bayesian phylogenetic analysis (Fig 2). The Indian golden jackal haplotypes clustered in a shallow monophyletic group, distinct from related wolf and domestic dog lineages in the Indian subcontinent (Fig 2). Average Tamura-Nei + G genetic distances between the golden jackal and other *Canis* sp. showed high divergence at both cyt *b* (>5%) and CR (>10%) sequences (Table 3). The taxa closest to *C*. *aureus* are Ethiopian wolf (*C*. *simiensis*, 5.3% and 19%), coyote (*C*. *latrans*, 5.8% and 18.9%), Himalayan wolf (*C*. *l*. *chanco*, 5.3% and 12.9%) and peninsular Indian wolf (*C*. *l*. *pallipes*, 5.6% and 16.5%), based on cyt *b* and CR sequences respectively. Average cyt *b* and CR distances between golden jackal and the wolf-dog clade was slightly higher (8.2% and 15.1% respectively). Besides these relationships to previously recognized wolves from various parts of the globe, we also compared sequences of canids recently proposed to represent African wolves (*C*. *lupus lupaster*, 5.2% and 17.2% for cyt *b* and CR respectively) which were previously classified as golden jackals [11].

**Table 1:** Control Region (CR) haplotypes and variable sites in golden jackals. Numbers above each nucleotide base represent nucleotide positions relative to the complete mtDNA genome of *Canis lupus lupus* (GenBank Accession ID—NC009686). Sampled Indian localities are Uttar Pradesh (UP), Uttarakhand (UA), Haryana (HR), Rajasthan (RJ), Gujarat (GJ), Madhya Pradesh (MP) and Karnataka (KR). Refer S1 Table for specific sample and locality information.

Haplotype ID	1	1	1	1	1	1	1	1	1	1	1	1	1	1	1	1	1	1	1	1	1	1	1	1	1	1	1	Locality (*n*)
	5	5	5	5	5	5	5	5	5	5	5	5	5	5	5	5	5	5	5	5	5	5	5	5	5	5	5	
	4	4	4	4	4	5	5	5	5	5	5	5	5	5	6	6	6	6	6	6	6	6	6	6	7	7	7	
	6	6	8	9	9	1	1	2	2	2	3	3	3	8	1	1	1	2	2	3	3	3	3	5	1	5	5	
	5	5	5	4	8	0	8	7	8	8	0	1	6	6	1	3	9	1	9	3	4	6	9	4	1	0	1	
Ind1	C	-	A	G	T	T	A	C	-	-	C	T	T	A	G	T	A	T	C	T	T	A	C	**A**	C	A	C	GJ (2); UP(3); RJ (3)
Ind2	C	**C**	A	G	**C**	T	-	**T**	-	-	C	**C**	T	A	G	T	A	T	C	T	T	A	C	G	C	A	C	GJ (4)
Ind3	C	**C**	A	G	**C**	T	-	**T**	**T**	**T**	C	**C**	**C**	A	G	T	A	T	C	T	T	A	C	G	C	A	C	GJ (4)
Ind4	C	-	A	G	T	T	A	C	-	-	C	T	T	A	G	T	A	T	C	T	T	A	C	G	C	A	**T**	UP(1)
Ind5	C	-	A	G	T	**C**	-	C	-	-	C	T	T	A	G	T	A	T	C	T	T	A	C	G	**T**	A	C	GJ (16)
Ind6	C	-	A	**A**	T	T	A	C	-	-	C	T	T	**G**	G	T	A	T	C	T	T	A	C	**A**	**T**	A	C	UP (1)
Ind7	C	-	A	G	T	T	A	C	-	-	C	T	T	A	G	T	A	T	C	T	T	A	C	**A**	**T**	A	C	HR (2)
Ind8	C	**C**	A	G	T	T	-	C	-	-	C	T	T	A	G	T	A	T	C	T	T	A	C	G	**T**	A	C	GJ (2); UA (1)
Ind9	C	-	A	G	T	T	A	C	-	-	C	T	T	A	G	**C**	A	T	C	T	**C**	A	C	G	C	A	C	KR (3)
Ind10	C	-	A	G	T	T	A	C	-	-	C	T	T	**G**	**A**	T	A	T	C	T	T	A	C	G	C	A	C	MP (2)
Ind11	C	-	A	G	T	T	A	C	-	-	C	T	T	A	G	T	A	T	C	T	T	A	C	G	C	A	C	UP (1)
Ind12	C	-	A	G	T	T	-	C	-	-	**T**	**C**	T	A	G	T	A	T	**T**	T	T	A	C	**A**	C	A	C	UP (1)
Ind13	C	-	A	G	T	T	A	C	**C**	-	C	T	T	A	G	T	**G**	T	C	T	T	A	C	G	C	A	**T**	UP (1)
Ind14	C	-	**G**	G	T	T	A	C	-	-	C	T	T	A	G	T	A	T	C	T	T	A	**T**	**A**	**T**	A	C	MP (1)
Ind15	C	-	A	G	T	T	A	C	-	-	C	T	T	**G**	G	T	A	T	C	T	T	A	C	G	**T**	A	C	UA (1); UP (1)
Ind16*	-	-	A	G	T	T	A	C	-	-	C	T	T	A	G	T	A	T	C	**C**	T	**G**	C	**A**	C	A	C	Central India (2)
Isr	C	-	A	G	T	T	A	C	-	-	C	T	T	**G**	G	T	A	**C**	C	T	T	A	C	**A**	**T**	A	C	Israel (2)
Eur**	C	-	A	G	T	T	A	C	-	-	C	T	T	**G**	G	T	A	T	C	T	T	A	C	G	**T**	**G**	C	Bulgaria (60); Serbia (129); Croatia (50); Italy (7); Austria (1)

* CR haplotypes reported in Aggarwal et al. [39], and

** in Zachos et al. [7], Fabbri et al. [9] and Randi et al. [38]

**Table 2:** Variable sites and haplotype frequency at the cytochrome *b* (cyt *b*) gene in golden jackals. Numbers above each nucleotide base represent nucleotide positions relative to the complete mtDNA genome of *Canis lupus lupus* (GenBank Accession ID—NC009686). Sampled Indian localities are Uttar Pradesh (UP), Uttarakhand (UA), Haryana (HR), Rajasthan (RJ), Gujarat (GJ), Madhya Pradesh (MP) and Karnataka (KR). Refer S2 Table for specific sample and locality information.

Haplotype ID	14,279	14,289	14,319	14,334	14,352	14,360	14,393	14,571	14,669	Locality (*n*)
cytb_Ind01	T	T	C	T	T	G	T	**A**	G	India—UP (6); UA (1); HR (2); RJ (2)
cytb_Ind02	T	T	C	T	T	G	T	G	G	India—GJ (3); UP (2); MP (2)
cytb_Ind03	T	T	C	T	**C**	G	T	G	G	India—GJ (6)
cytb_Ind04	T	T	C	T	T	G	T	G	**A**	India—KR (2)
cytb_Ind05	T	T	C	T	T	G	**C**	G	G	India—GJ (3)
cytb_Ind06	T	**C**	C	T	T	G	T	G	G	India—UA (2)
cytb_Ind07*	T	T	**T**	T	T	**A**	T	G	G	Central India (2)
cytb_Isr	T	T	C	**C**	T	G	T	G	G	Israel (2)
cytb_Blg	**A**	T	C	**C**	T	G	T	G	G	Bulgaria (5)

* haplotype reported in Aggarwal et al.[39]

**Table 3:** Mean Tamura-Nei + G genetic distances among the main *Canis* lineages and other wolf-like canids, estimated from cytochrome *b* (below diagonal) and control region (above diagonal) sequences. Values in square brackets represent standard error estimates. Depicted taxa include golden jackal (GJ), African wolf (AW), wolf-dog clade (WDC), Indian wolf (IW), Himalayan wolf (HW), coyote (CY), Ethiopian wolf (EW), black-backed jackal (BBJ), side-striped jackal (SSJ), dhole (DL), African wild dog (AWD) and maned wolf (MW). Distance not estimated (ne) as CR sequence of BBJ is not available.

	GJ	AW	WDC	IW	HW	CY	EW	BBJ	SSJ	DL	AWD	MW
**GJ**		0.172 [0.054]	0.151 [0.053]	0.165 [0.066]	0.129 [0.045]	0.184 [0.066]	0.190 [0.063]	ne	0.285 [0.110]	0.254 [0.076]	0.436 [0.175]	0.198 [0.057]
**AW**	0.052 [0.016]		0.079 [0.024]	0.100 [0.032]	0.092 [0.030]	0.166 [0.038]	0.157 [0.048]	ne	0.204 [0.066]	0.232 [0.071]	0.280 [0.095]	0.142 [0.038]
**WDC**	0.082 [0.022]	0.054 [0.017]		0.054 [0.019]	0.087 [0.030]	0.100 [0.034]	0.153 [0.050]	ne	0.149 [0.049]	0.179 [0.053]	0.272 [0.098]	0.173 [0.046]
**IW**	0.056 [0.017]	0.031 [0.012]	0.018 [0.007]		0.091 [0.034]	0.110 [0.039]	0.175 [0.057]	ne	0.153 [0.050]	0.230 [0.078]	0.304 [0.117]	0.227 [0.070]
**HW**	0.053 [0.015]	0.027 [0.010]	0.045 [0.014]	0.023 [0.009]		0.162 [0.057]	0.178 [0.060]	ne	0.184 [0.059]	0.171 [0.049]	0.337 [0.131]	0.209 [0.064]
**CY**	0.058 [0.018]	0.058 [0.019]	0.076 [0.021]	0.052 [0.016]	0.049 [0.016]		0.192 [0.064]	ne	0.204 [0.079]	0.296 [0.105]	0.289 [0.099]	0.224 [0.070]
**EW**	0.053 [0.018]	0.054 [0.019]	0.072 [0.021]	0.048 [0.016]	0.053 [0.017]	0.040 [0.014]		ne	0.210 [0.067]	0.302 [0.098]	0.268 [0.092]	0.237 [0.074]
**BBJ**	0.139 [0.036]	0.140 [0.044]	0.174 [0.046]	0.154 [0.043]	0.118 [0.034]	0.151 [0.041]	0.114 [0.031]		ne	ne	ne	Ne
**SSJ**	0.200 [0.058]	0.223 [0.075]	0.288 [0.090]	0.271 [0.090]	0.243 [0.081]	0.249 [0.083]	0.182 [0.053]	0.216 [0.070]		0.223 [0.077]	0.266 [0.097]	0.313 [0.120]
**DL**	0.181 [0.052]	0.170 [0.057]	0.184 [0.050]	0.165 [0.049]	0.144 [0.047]	0.181 [0.053]	0.198 [0.065]	0.193 [0.064]	0.222 [0.073]		0.376 [0.155]	0.258 [0.084]
**AWD**	0.311 [0.116]	0.292 [0.118]	0.402 [0.160]	0.340 [0.136]	0.356 [0.155]	0.322 [0.122]	0.319 [0.126]	0.268 [0.095]	0.283 [0.100]	0.208 [0.072]		0.377 [0.165]
**MW**	0.202 [0.058]	0.181 [0.155]	0.250 [0.073]	0.202 [0.060]	0.185 [0.059]	0.215 [0.069]	0.222 [0.071]	0.272 [0.084]	0.407 [0.149]	0.258 [0.087]	0.305 [0.100]	

**Fig 3:**
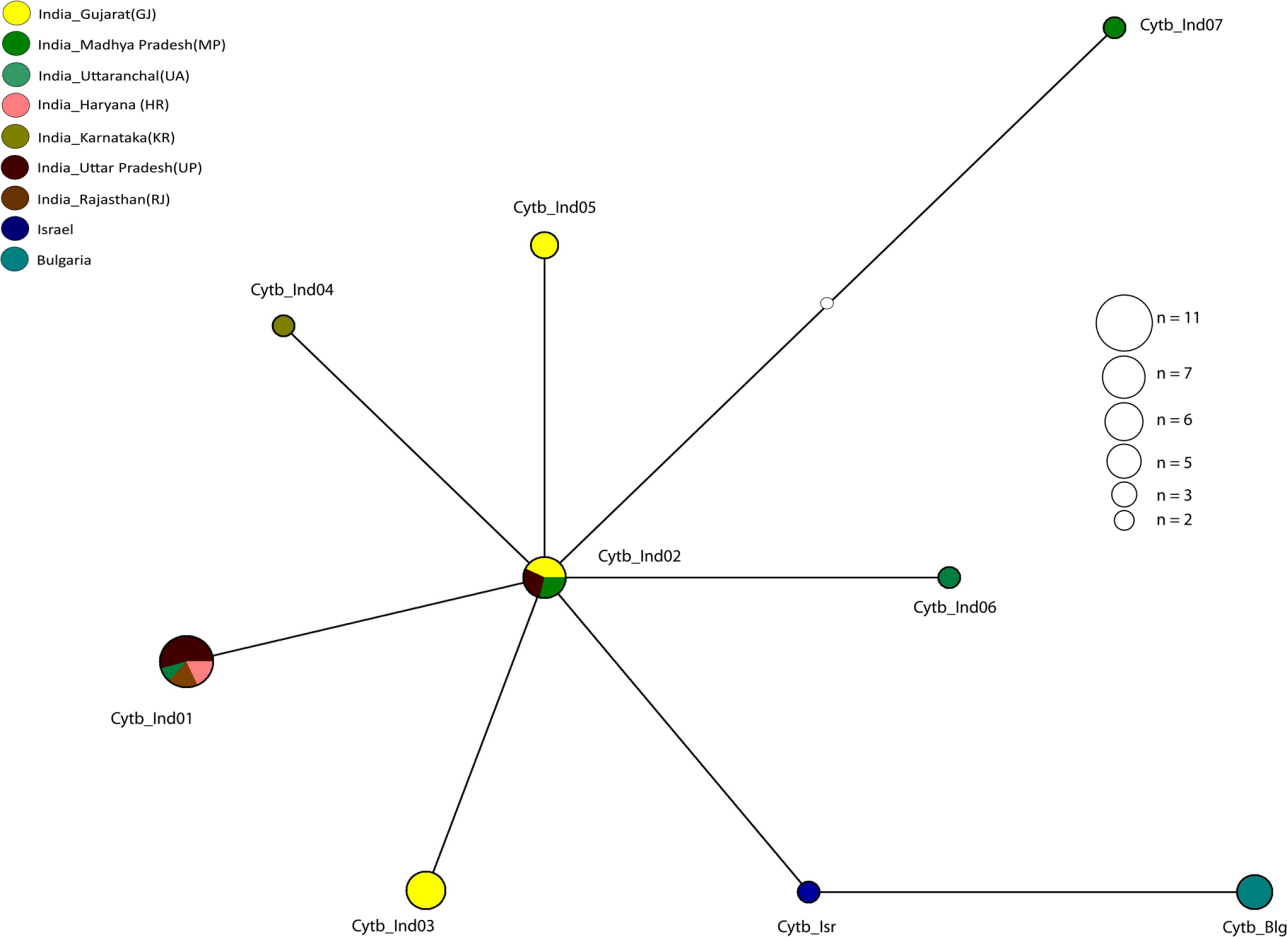
Median joining mtDNA cyt *b* (390 bp) haplotype network tree, showing star-like radiation and polytomy of golden jackal haplotypes. Haplotype circles are colour coded according to geographic locality, and circle size is proportional to haplotype frequency. Each node represents a one base pair change. See Table 1 for variable nucleotide positions used in network construction. Circle sizes are proportional to the number of individuals represented by each haplotype.

**Fig 4:**
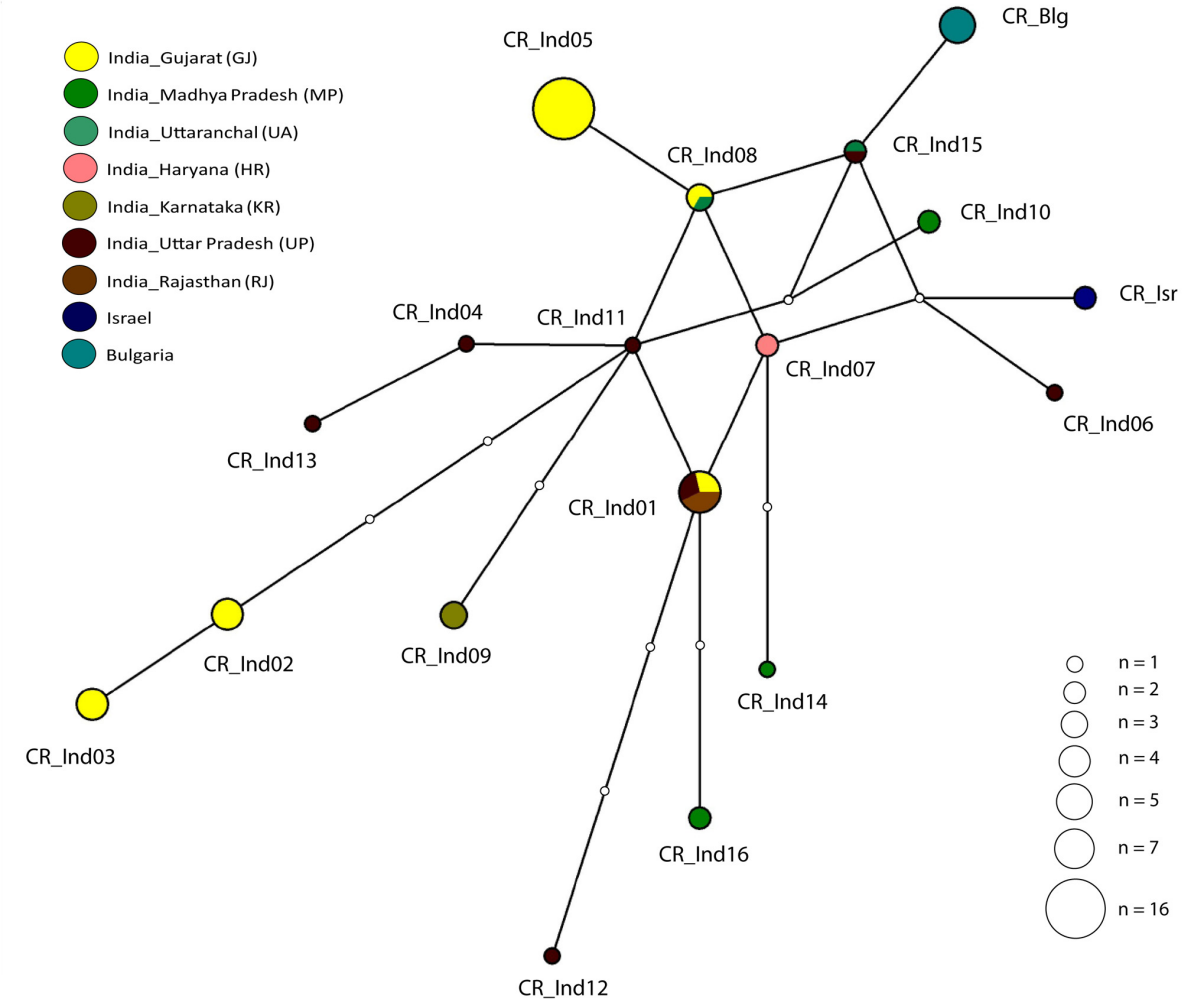
Median joining network of mtDNA CR (286 bp) haplotypes in golden jackals. Haplotype circle sizes are proportional to frequency, and colour coded according to geographic sampling area. Each node depicts a one base pair change. Variable nucleotide positions used in network construction are shown in Table 2. Circle sizes are proportional to the number of individuals represented by each haplotype.

The median joining cyt *b* haplotype network showed a shallow star-like radiation, and with individual haplotypes differing from each other by one to two nucleotide bases (Fig 3). On the other hand, the CR haplotypes revealed a weakly structured haplotype network with multiple alternative connections, and with haplotypes being separated from one another by one to three base substitution events (Fig 4). A similar pattern was also visible in the network analysis of the concatenated cyt *b* and CR haplotype sequences (S2 Fig). However, unlike the predominantly single base separation of individual cyt *b* and CR haplotypes, most haplotypes were separated from neighboring haplotypes by at least two or more base substitutions. Sixty one percent (11 of 18) of the CR haplotypes are completely peripheral with only one connection to the network (Fig 4). All interior haplotypes were represented by Indian samples. Haplotypes from Bulgaria and Israel were peripheral compared to Indian haplotypes and separated from Indian jackals by one and two base substitutions respectively (Fig 4). The north Indian CR haplotype CauInd11, represented by one jackal sampled in Uttar Pradesh, was the most interior in the network with six connections to other haplotypes (Fig 4). Haplotypes CauInd01, CauInd07, CauInd08 and CauInd15 obtained from golden jackal samples in northern and western India, each had four connections to the remaining haplotypes in the network (Fig 4). Although the phylogenetic trees and median joining network revealed a lack of genetic structure in Indian golden jackal, geographic division in haplotype composition was clearly observed between the southern and northern regions (Fig 1). CauInd01, which was represented by two jackals in Gujarat in western India, and three jackals each in Uttar Pradesh and Rajasthan in northern India (Fig 4), was clearly the most common haplotype in India and not observed in central and southern India (Fig 1). A few regional haplotypes included CauInd02 and CauInd05 in western India; CauInd07 in northern India; CauInd10, CauInd14 and CauInd16 in central India; and CauInd09 in Karnataka in southern India (Figs 1 and 4). The Bulgarian CR haplotype sequenced from five jackals in this study was identical to a previously published monomorphic European haplotype [7, 9, 38] obtained from 242 jackal samples in the Balkans (Fig 1).

**Fig 5:**
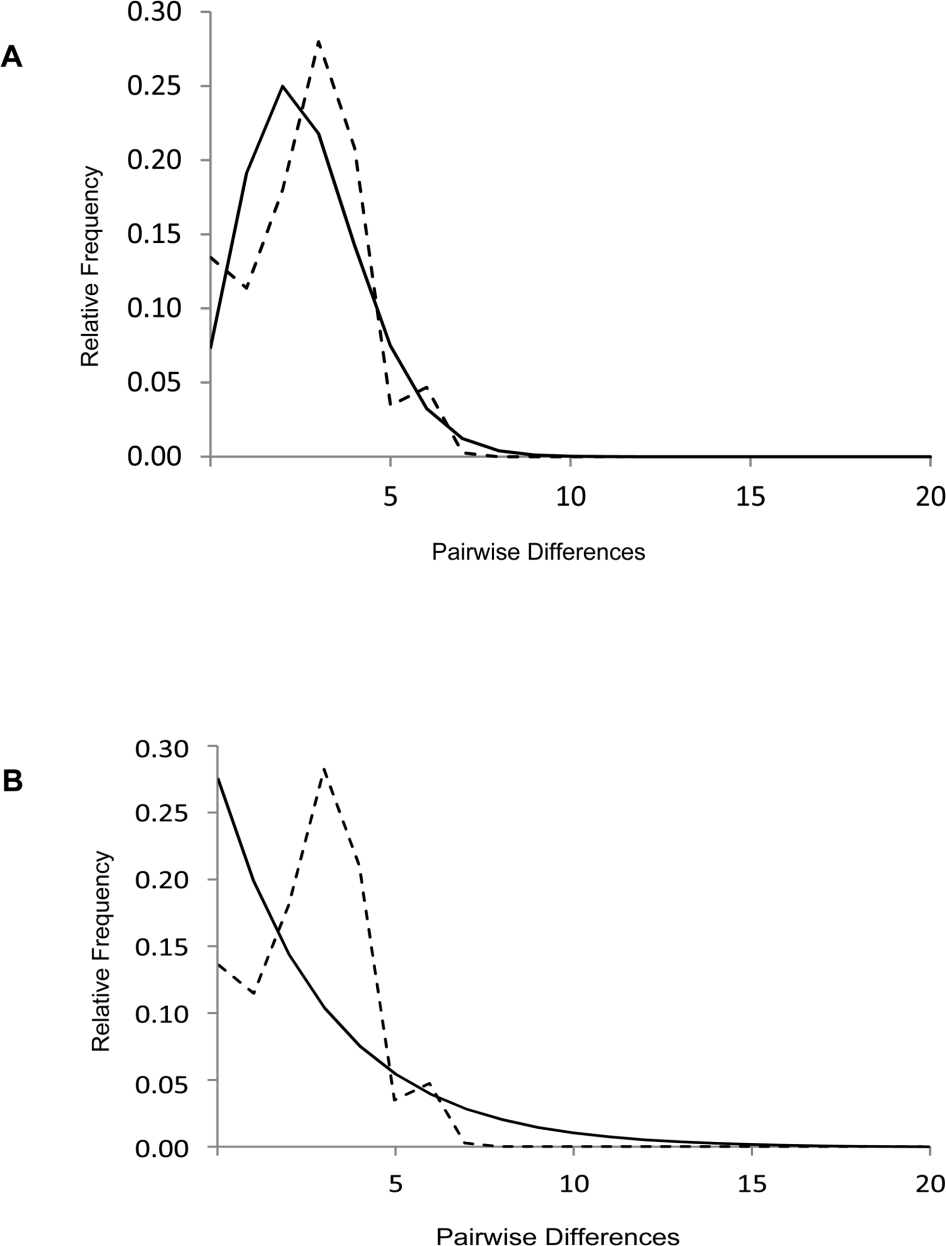
Mismatch distributions of pairwise differences of CR haplotypes for the golden jackal in India. Depicted are observed (dashed lines) and expected (solid lines) frequencies obtained under a model allowing (A) and not allowing for demographic expansion (B).

Golden jackals from India had the highest nucleotide and haplotype diversity compared to jackals from Israel or Bulgaria, or with other related wild *Canis* sp. from the Indian sub-continent (Table 4). The diversity observed in golden jackals is comparable to domestic dog CR sequences reported in a previous study [23]. Observed values for mismatch distribution of pairwise differences among all Indian jackal haplotypes produced a right-skewed unimodal peak which is characteristic of demographic expansion (Fig 5). The mismatch distribution was highly informative because of the high genetic diversity of jackals within India. It was not informative for Europe and Israel due to the extremely low haplotype diversity. The distribution of observed mismatch values produced a good fit with the expected distribution as seen in the low Harpending’s raggedness index in Indian jackals (*r* = 0.045) compared to golden jackals from other regions and related Indian canids (Table 5). Tajima’s *D* and Fu’s *F*_*S*_ statistics were negative in golden jackal, indicative of population expansion or selection in the species, and values were relatively higher compared to Indian and Himalayan wolf, and domestic dog lineages (Table 5). We used the estimate of 5.414 for *Τ* obtained from the mismatch distributions for jackals and a mutation rate *μ* of 7.30 x 10^−5^ per generation as previously applied in gray [35] and red foxes [36]. By rearranging the formula *Τ = 2μt*, we estimated the time since expansion (*t*) of ~37,083 years before present (YBP) for the Indian jackals (Table 5).

**Table 4:** Summary statistics of molecular diversity at mtDNA control region in golden jackal and related Indian canids.

Species	*n*	No. of segregating sites (*S*)	No. of haplotypes	Haplotype diversity (*h*) ± Std. Dev.	Nucleotide diversity (*Π*) ± Std. Dev.	Mean pairwise differences (*k*)
*C*. *aureus* (India)	51	25	16	0.8678 ± 0.0326	0.0162 ± 0.0088	5.091
*C*. *aureus* (Israel, Europe)	249	3	2	0.0160 ± 0.0111	0.0002 ± 0.0004	0.048
*C*. *aureus* (Eurasia)	300	20	18	0.3444 ± 0.0352	0.0059 ± 0.0038	1.861
*C*. *lupus pallipes*	45	4	4	0.4929 ± 0.0658	0.0024 ± 0.0021	0.685
*C*. *l*. *chanco*	16	8	5	0.5333 ± 0.1421	0.0042 ± 0.0032	1.200
*C*. *l*. *familiaris*	24	17	11	0.8986 ± 0.0370	0.0147 ± 0.0082	4.996

**Table 5:** Demographic expansion parameters estimated from control region sequences, for golden jackal and related wolf and feral dog lineages in India. *Τ* is the coalescent time of expansion.

Species	Tajima's *D* (*P*)		Fu's *Fs* (*P*)		Raggedness statistic, *r*	*Τ*	Expansion time estimated from *Τ* in YBP (90% C.I.)
*C*. *aureus* (India)	-0.806	0.250	-1.351	0.342	0.045	5.414	37,083 (16,133–71,249)
*C*. *aureus* (Israel, Europe)	-1.401	0.021	-1.087	0.094	0.969	3.000	20,548 (20,548–20, 548)
*C*. *aureus* (Eurasia)	-1.592	0.016	-5.036	0.075	0.443	3.000	20,548 (2,649–23,973)
*C*. *lupus pallipes*	-0.81	0.263	-0.17	0.412	0.169	0.654	4,482 (2,528–7,103)
*C*. *l*. *chanco*	-1.811	0.010	-0.967	0.190	0.113	0.000	not estimated
*C*. *l*. *familiaris*	0.35	0.713	-0.999	0.328	0.041	8.486	58,126 (16,842–83,008)

## Discussion

In this study, we sequenced a 440 bp fragment of the noncoding mtDNA CR locus and 412 bp of the coding mtDNA cyt *b* gene to study the phylogeography of golden jackal in the Indian subcontinent. MtDNA is inherited from the female parent as a single linkage unit, and has a mutation rate that is five to ten times higher than the average rate of synonymous substitutions at single-copy protein nuclear genes in mammals [40]. Furthermore, variable segments within the non-coding CR segment evolve about four to five fold faster that the entire mtDNA molecule [40]. Hence, due to its high mutation rates, small size and conserved arrangement of genes, mtDNA is extensively used to study phylogeography across many vertebrate species [41]. The phylogenetic analysis of golden jackal and related *Canis* sp., including the African wild dog (*Lycaon pictus*), dhole (*Cuon alpinus*) and maned wolf as outgroups, supports similar relationships of this group of *Canis* as previously reported by Lindblad-Toh et al. [42], Rueness et al. [11] and Gaubert et al. [12]. Golden jackals from India, the Middle East and Europe formed a monophyletic clade, which was highly divergent from other canid species (cyt *b* genetic distance >5%). The cyt *b* gene is commonly used as a genetic marker to distinguish among mammalian species, and >5% sequence divergence is typically observed between morphologically recognized species [43]. Despite the high degree of morphological and behavioural similarity of black-backed (*C*. *mesomelas*) and side-striped jackals (*C*. *adustus*) to golden jackals [36], they are not closely related to each other (>10 to 20% cyt *b* genetic distances). The paraphyletic origins of the jackal group have also been reported by previous studies on canid systematics [10, 11, 12, 13, 42]. Although sequence divergence was high (>5% cyt *b* genetic distance), golden jackals formed a sister clade with the Ethiopian wolf, albeit with low phylogenetic support (Bayesian posterior probability < 0.90). Phylogenetic support was similarly low (Bayesian posterior probability < 0.90) at the fork leading to the divergence of the archetypal wolf clades (gray, Indian, Himalayan wolf and domestic dog lineages) from the neighbouring clade represented by the coyote, Ethiopian wolf and golden jackal. However, the evolutionary relationships among these group of *Canis* species have been well established [10, 42] and the low nodal support values observed in our analysis is likely an artifact of using short DNA sequences for phylogenetic alignment in this study.

The phylogenetic analysis revealed a polytomy and no clear pattern of genetic structure between Indian, Israeli, Bulgarian and previously published haplotypes (Fig 2). We excluded African golden jackal haplotypes from Kenya [10] in our analyses due to the questionable nature of these sequences as highlighted by Rueness et al. [11] and Gaubert et al. [12], which revealed these sequences to closely resemble the side-striped jackal (*Canis adustus*) and not golden jackal. Unlike the noticeable incidences of hybridization between golden jackal and gray wolf observed in the African golden jackal/ wolf (*Canis lupus (aureus) lupaster*) in western and northern Africa [11, 12] and to a lesser extent in Europe [14], there was no evidence in our data to suggest mtDNA gene flow between the golden jackal and with other related Indian, Himalayan and wolf-dog clades in India. Although there is some indication that golden jackals can breed with domestic dogs in captivity [44] and anecdotal accounts exist of Indian feral dogs which are strikingly similar to jackals [45], we detected no evidence of hybridization in our study. However, our sampling was opportunistic and limited in scale for a conclusive study on hybridization between the different *Canis* species in India. The ranges of golden jackal and gray wolf overlap greatly not just in India, but also in southern Asia, the Middle East and the Balkans. Hybridization between jackals and wolves (and presumably feral dogs as well) may occur, especially in localities where population depletion has led to reduced mate availability in the wild. Extensive sampling of golden jackals, wolves and feral dogs, especially around areas of ecological overlap and contact zones from throughout the Indian subcontinent, is required to detect any incidence of potential admixture in this region. Furthermore, information from bi-parentally inherited nuclear markers is required as mtDNA sequencing can only reveal maternal genetic history. Further studies using autosomal nuclear markers (microsatellites and SNPs) and Y-chromosome linked markers are needed to evaluate the population structure, demographic history and potential hybridization with other canid species in the region.

Our results show that golden jackal populations in India exhibit high haplotype diversity, with no clear phylogeographic breaks and likely have undergone recent demographic expansion. Six of the seven cyt *b* haplotypes and fifteen of sixteen CR haplotypes found in India were novel and have not yet been reported for other golden jackals published in GenBank. No clear phylogeographic pattern in India could be inferred from analysis of cyt *b* and CR haplotypes. Since cyt *b* had low resolution and revealed a polytomy as evidenced by the shallow star-like radiation in the median-joining network (Fig 3), we attempted to resolve the phylogeographic patterns using the more variable CR fragment of the mtDNA genome. In the few instances where CR haplotypes were shared between localities, they were not always shared with jackals from the same geographic region (Figs 1 and 4) and the level of resolution of the phylogenetic tree did not reveal any clear phylogeographic breaks (Fig 2). Lineage interpretation was confounded by multiple alternative connections across some haplotypes that are equally parsimonious, which could be due to homoplasy caused by the high mutation rate of the control region [41] and rapid expansion of the golden jackal populations due to high dispersal ability [3, 4]. In contrast, the cyt *b* network showed a clear radiation of European and Middle Eastern haplotypes from India, compared to the intermingled CR network.

Assuming that the ancestral haplotypes are internal while the derived haplotypes are peripheral, the CR haplotype CauInd11 with six connections to other haplotypes, and CauInd01, CauInd07, CauInd08 and CauInd15 with four connections, are relatively ancestral compared to the remaining haplotypes. These basal haplotypes were restricted to western and northern India and absent from other sampled regions. In contrast, CR haplotypes sampled from southern (CauInd09) and central India (CauInd10, CauInd14 and CauInd16) in our study were observed to be localized and more derived relative to northern and western Indian haplotypes. The most widespread Indian haplotype is CauInd01, represented by eight individuals from western (Gujarat, Rajasthan) and northern (Uttar Pradesh) India. CauInd11 with six connections is the most ancestral haplotype sampled in our study. Due to low sampling intensity across India (except Gujarat state in western India) it is quite likely that more ancestral haplotypes may as yet be unsampled. The evidence of extensive branching, existence of alternative connections and shallow divergence seen in the golden jackal haplotype network is indicative of a population that has recently undergone demographic expansion. Indian CR haplotypes are not monophyletic with respect to other haplotypes found in GenBank [7, 39] and sequenced from Israel and Europe in this study. The high haplotype diversity at the CR locus for Indian golden jackal populations contrasts with the monomorphic CR haplotype consistently observed in Bulgarian and neighbouring Balkan populations in south-eastern Europe [7, 9]. Our findings of high haplotype diversity in Indian golden jackals dispel the notion of mtDNA CR monomorphism in European populations having arisen from exceptionally conserved molecular dynamics, but rather from a signature of recent expansion in the area [9]. The dominant presence of this monomorphic haplotype in Europe could have resulted from a few long-distance dispersers at the leading edge of an expansion event which colonize a new area, leading to modern signals of reduced genetic diversity [46]. A similar pattern is also observed in the widespread Himalayan wolf (*C*. *lupus chanco*) mtDNA haplotype C lineage which is present in a wide-arc from Kashmir to eastern Nepal [23].

Signatures of demographic expansion or selection in the Indian golden jackal populations are evident from both Tajima’s *D* and Fu’s *F*_*S*_ test statistics which are negative and in the mismatch distribution of pairwise differences which produced a right-skewed unimodal peak characteristic of demographic expansion. The non-significance of observed test statistics is likely due to the low sample sizes across many haplotypes. By using estimates of mutation rate previously applied in related species [35, 36], to a simple coalescence equation, we timed the start of the demographic expansion seen in the Indian jackal populations to almost 37,083 YBP (90% CI: 16,133–71,249). Golden jackals from Europe, Israel and the entire Eurasian population (including India) were dated to expand much later ~20,548 YBP (90% CI: 2,649–23,973). These expansion dates coincide with the estimated periods of the inter-glacial cycles of the late Pleistocene, wherein certain relatively warmer areas in India and south-east Asia were believed to have served as refugia during the last glacial maxima *~* 18 000 to 25,000 years ago [47, 48]. The presence of refugial populations in the Indian subcontinent during the Pleistocene glaciations is also corroborated by the existence of unique wolf lineages in the region which are basal and divergent from all other widespread wolf-dog clades [27]. However, it has been demonstrated that mtDNA polymorphism can be generated much before sister populations diverge [49], so our estimated time of expansion is probably an overestimate of the time of split. This could be the case with golden jackal colonization over many parts of Europe which appear to be of recent origin, although the estimate assumes that the present haplotypes have evolved in the region 20,458 years ago to the observed composition and frequency. It is more likely that the European populations harboring this unique haplotype at the northern extremity of golden jackal range, evolved from southern refugial populations which later expanded into the region. Records suggest that much of the sampled European populations were founded by migrants in recent decades, owing to habitat related changes and the extirpation of wolves from the area [4, 9].

Our study showed that haplotype diversity in the golden jackal was much higher than in any other member of the genus *Canis* surveyed to date in India. Only four CR haplotypes were recovered for peninsular Indian wolf, five for Himalayan wolf and eleven for *C*. *lupus familiaris* [27]. In addition, only one cyt *b* haplotype was found when sequencing multiple individuals from different localities for each of these species [27]. However, cyt *b* sequences of Indian jackals revealed seven different but closely related haplotypes throughout the region, only differing by one or two base substitutions from one another (Fig 3 and Table 1). This suggests that golden jackals have high levels of mtDNA diversity compared to all the other *Canis* sp. that inhabit this part of the world including feral and domestic dogs. It also suggests that historically golden jackals have had large effective population sizes and a potentially longer evolutionary history in India than in other parts of the world, although further sampling from Africa, the Middle East and south-east Asia is needed to test this hypothesis. Past geological events in the Indian sub-continent may have shaped the current phylogeographic distribution of golden jackals that we observe today. However, current understanding of golden jackal evolutionary history based on genetic data alone, as presented in this study cannot predict a narrow enough time frame for expansion as it encompasses a wide interval (16,133 to 71,249 YBP). The post-Pleistocene expansion from refugial populations in the Indian-subcontinent to neighbouring regions seems likely as attested by the negative Tajima’s and Fu’s test statistics and right-skewed mismatch distribution peak. The star shaped polytomy of the haplotype network suggest that golden jackals may have undergone dramatic demographic changes in the recent past. Based on currently available data the Indian-subcontinent seems to be the center of radiation of golden jackals.

## Supporting Information

S1 Fig Map showing sampling locations in India. (TIF)

S2 Fig Median joining haplotype network tree constructed from concatenated cyt *b* and CR sequences. Haplotype circles are colour coded according to geographic locality, and circle size is proportional to haplotype frequency. Each node represents a one base pair change. Circle sizes are proportional to the number of individuals represented by each haplotype.
(TIF)

S1 Table Control Region sequences of golden jackals sampled from India. (DOCX)

S2 Table Cytochrome b sequences of golden jackals sampled from India. (DOCX)

S3 Table Best model selection. Estimating the best model of molecular substitution inferred using Log Bayes Factors (LBF) from Bayesian posterior distributions in Mr Bayes. Rate variation for tested models—gamma distributed rate variation across sites (gamma); gamma distributed with proportion of invariable sites (invgamma); rate variation with proportion of invariable sites (propinv); equal rate variation across sites (equal).
(DOCX)
