# Supplementary figures and images for: Phylogeography of the Golden Jackal (Canis aureus) in India
Source: PLoS One. 2015 Sep 28;10(9):e0138497. doi: 10.1371/journal.pone.0138497 (PMC4586146; doi:10.1371/journal.pone.0138497)

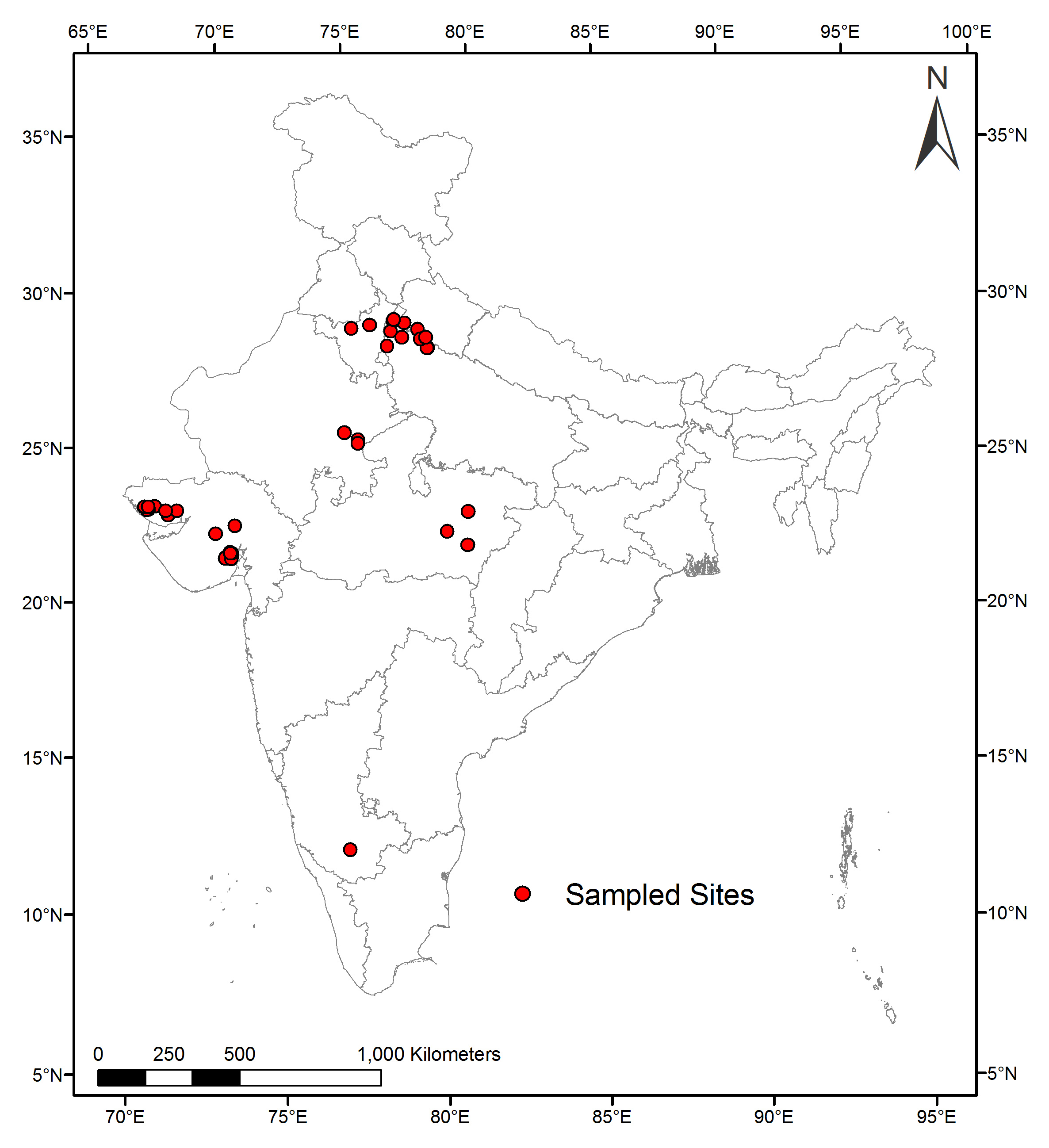

Supplement: S1 Fig — (TIF) [file pone.0138497.s001.tif]

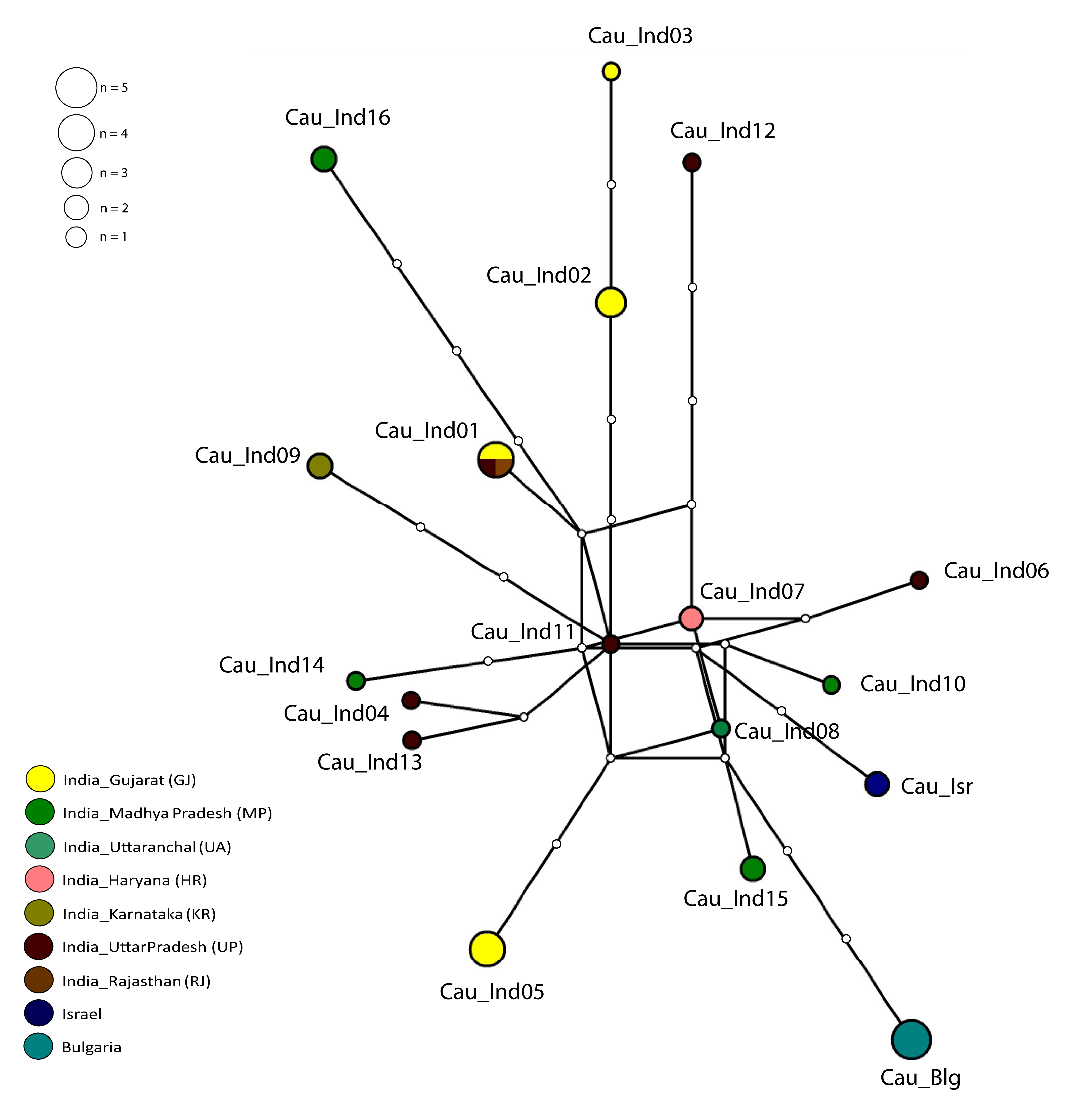

Supplement: S2 Fig — Haplotype circles are colour coded according to geographic locality, and circle size is proportional to haplotype frequency. Each node represents a one base pair change. Circle sizes are proportional to the number of individuals represented by each haplotype. (TIF) [file pone.0138497.s002.tif]
