# Supplementary material for: Phylogeography of the Golden Jackal (Canis aureus) in India
Source: PLoS One. 2015 Sep 28;10(9):e0138497. doi: 10.1371/journal.pone.0138497 (PMC4586146; doi:10.1371/journal.pone.0138497)
Supplement: S1 Table — (DOCX) [file pone.0138497.s003.docx]

**S1 Table. Control Region sequences of golden jackals sampled from India**

| ***Specimen ID*** | ***Haplotype*** | ***Genbank Acc. #*** | ***Sample type*** | ***Latitude (N)*** | ***Longitude (E )*** | ***Locality*** | ***Date*** |
| --- | --- | --- | --- | --- | --- | --- | --- |
| D269 | CR_Ind01 | KT343787 | Hair | 29.0352 | 78.7574 | Moradabad, Uttar Pradesh | 01 October 2005 |
| D29b | CR_Ind01 | KT343787 | Tissue | 21.9042 | 71.8784 | Vallabhipur, Bhal, Gujarat | 06 January 1996 |
| D31 | CR_Ind01 | KT343787 | Tissue | 23.2956 | 70.0596 | Hirapal, Kachchh, Gujarat | 01 June 1997 |
| D471 | CR_Ind01 | KT343787 | Tissue | 26.1878 | 75.8373 | Tonk, Rajasthan | 26 December 2007 |
| D476 | CR_Ind01 | KT343787 | Tissue | 25.9779 | 76.3318 | Bakija, Ranthambore Tiger Reserve, Rajasthan | 11 February 2007 |
| D477 | CR_Ind01 | KT343787 | Tissue | 25.8618 | 76.3375 | Ranthambore Tiger Reserve, Rajasthan | 20 December 2007 |
| D480 | CR_Ind01 | KT343787 | Tissue | 29.0584 | 77.2669 | Badoth, Uttar Pradesh | 20 February 2008 |
| D268 | CR_Ind01 | KT343787 | Hair | 29.9305 | 77.4899 | Saharanpur, Uttar Pradesh | 13 November 2005 |
| D152 | CR_Ind02 | KT343788 | Blood | 22.0514 | 72.0378 | Velavadar National Park, Gujarat | 14 November 2000 |
| D156 | CR_Ind02 | KT343788 | Tissue | 22.0583 | 72.0244 | Velavadar National Park, Gujarat | 14 November 2000 |
| D167 | CR_Ind02 | KT343788 | Blood | 22.0596 | 72.0571 | Velavadar National Park, Gujarat | 19 December 2000 |
| D41 | CR_Ind02 | KT343788 | Tissue | 23.3700 | 69.2393 | Kachchh, Gujarat | 23 January 1997 |
| D158 | CR_Ind03 | KT343789 | Tissue | 22.0555 | 72.0493 | Velavadar National Park, Gujarat | 14 November 2000 |
| D26c | CR_Ind03 | KT343789 | Tissue | 23.3335 | 70.3459 | Kachchh, Gujarat | 23 January 1997 |
| D556 | CR_Ind03 | KT343789 | Blood | 23.3334 | 69.0317 | Kachchh, Gujarat | 01 December 1999 |
| D84 | CR_Ind03 | KT343789 | Tissue | 21.8756 | 72.0890 | Vegad, Bhal, Gujarat | 22 February 1996 |
| D470 | CR_Ind04 | KT343790 | Tissue | 29.3859 | 78.6780 | Afzalgarh, Uttar Pradesh | 11 September 2007 |
| D130 | CR_Ind04 | KT343790 | Tissue | 23.2949 | 70.0487 | Kachchh, Gujarat | 05 January 1997 |
| D44 | CR_Ind04 | KT343790 | Tissue | 23.1217 | 69.7574 | Kachchh, Gujarat | 24 January 1997 |
| D150 | CR_Ind05 | KT343791 | Blood | 22.0557 | 72.0226 | Velavadar National Park, Gujarat | 13 November 2000 |
| D153 | CR_Ind05 | KT343791 | Blood | 22.0604 | 72.0428 | Velavadar National Park, Gujarat | 15 February 1995 |
| D155 | CR_Ind05 | KT343791 | Tissue | 22.0642 | 72.0392 | Velavadar National Park, Gujarat | 16 November 2000 |
| D168 | CR_Ind05 | KT343791 | Blood | 22.0743 | 72.0325 | Velavadar National Park, Gujarat | 20 December 2000 |
| D172 | CR_Ind05 | KT343791 | Blood | 22.0428 | 72.0384 | Velavadar National Park, Gujarat | 28 December 2000 |
| D30 | CR_Ind05 | KT343791 | Blood | 21.9516 | 72.0971 | Bhal, Gujarat | 02 December 1996 |
| D34 | CR_Ind05 | KT343791 | Tissue | 22.0413 | 72.0292 | Velavadar National Park, Gujarat | 01 January 1995 |
| D35 | CR_Ind05 | KT343791 | Tissue | 22.0383 | 72.0288 | Velavadar National Park, Gujarat | 05 June 1995 |
| D36 | CR_Ind05 | KT343791 | Tissue | 22.0563 | 72.0708 | Bhal, Gujarat | 17 June 1995 |
| D38 | CR_Ind05 | KT343791 | Tissue | 23.2984 | 68.9222 | Kachchh, Gujarat | 23 September 1997 |
| D39 | CR_Ind05 | KT343791 | Blood | 22.9485 | 72.1319 | Vegad, Bhal, Gujarat | 22 February 1996 |
| D40 | CR_Ind05 | KT343791 | Hair | 23.3064 | 68.8895 | Kachchh, Gujarat | 23 January 1997 |
| D42 | CR_Ind05 | KT343791 | Tissue | 21.8739 | 71.8736 | Vallabhipur, Bhal, Gujarat | 06 January 1997 |
| D43 | CR_Ind05 | KT343791 | Blood | 23.2538 | 69.0536 | Kachchh, Gujarat | 24 January 1997 |
| D45 | CR_Ind05 | KT343791 | Tissue | 23.2413 | 68.9932 | Kachchh, Gujarat | 23 January 1997 |
| D46 | CR_Ind05 | KT343791 | Tissue | 21.9720 | 72.1118 | Bhal, Gujarat | 21 February 1996 |
| D150b | CR_Ind05 | KT343791 | Blood | 22.0557 | 72.0226 | Velavadar National Park, Gujarat | 13 November 2000 |
| D230 | CR_Ind05 | KT343791 | Tissue | 22.6393 | 71.4645 | Muli road, Gujarat | 24 March 2001 |
| D270 | CR_Ind06 | KT343792 | Hair | 29.0407 | 78.7468 | Moradabad, Uttar Pradesh | 01 November 2005 |
| D246 | CR_Ind07 | KT343793 | Tissue | 29.7081 | 76.5832 | Kaithal, Haryana | 05 May 2003 |
| D247 | CR_Ind07 | KT343793 | Tissue | 29.5952 | 75.9163 | Kaithal, Haryana | 06 May 2003 |
| D240 | CR_Ind08 | KT343794 | Tissue | 23.3334 | 68.9045 | Kachchh, Gujarat | 11 June 2002 |
| D424 | CR_Ind08 | KT343794 | Blood | 23.2600 | 69.6454 | Hyena Ridge, Kachchh, Gujarat | 01 July 2007 |
| D468 | CR_Ind08 | KT343794 | Tissue | 29.6338 | 78.3866 | Nazimabad, Uttar Pradesh | 11 November 2007 |
| D475-i | CR_Ind09 | KT343795 | Hair | 12.6715 | 76.6251 | Melkote, Karnataka | 01 April 2012 |
| D475-ii | CR_Ind09 | KT343795 | Hair | 12.6715 | 76.6251 | Melkote, Karnataka | 02 April 2012 |
| D475-iii | CR_Ind09 | KT343795 | Hair | 12.6715 | 76.6251 | Melkote, Karnataka | 03 April 2012 |
| D473 | CR_Ind10 | KT343796 | Tissue | 23.7369 | 80.3275 | Mandla-Jabalpur highway, Madhya Pradesh | 11 September 2007 |
| D474 | CR_Ind10 | KT343796 | Tissue | 23.0776 | 79.6165 | Mandla-Jabalpur highway, Madhya Pradesh | 12 September 2007 |
| D479 | CR_Ind11 | KT343797 | Tissue | 29.5331 | 77.3834 | Shamli, Uttar Pradesh | 02 October 2008 |
| D467 | CR_Ind12 | KT343798 | Tissue | 29.8904 | 77.4555 | Saharanpur, Uttar Pradesh | 13 November 2007 |
| D469 | CR_Ind13 | KT343799 | Tissue | 29.3282 | 78.4902 | Dhampur, Uttar Pradesh | 11 November 2007 |
| D257 | CR_Ind14 | KT343800 | Tissue | 22.6359 | 80.3404 | Mandla Road, Madhya Pradesh | 26 June 1995 |
| D248 | CR_Ind15 | KT343793 | Tissue | 29.8285 | 77.8647 | Roorkie, Uttarakhand | 01 November 2003 |
| D249 | CR_Ind15 | KT343801 | Hair | 29.3610 | 77.7949 | Muzzaffarnagar, Uttar Pradesh | 01 December 2003 |
