# Supplementary material for: Phylogeography of the Golden Jackal (Canis aureus) in India
Source: PLoS One. 2015 Sep 28;10(9):e0138497. doi: 10.1371/journal.pone.0138497 (PMC4586146; doi:10.1371/journal.pone.0138497)
Supplement: S2 Table — (DOCX) [file pone.0138497.s004.docx]

**S2 Table. Cytochrome *b* sequences of golden jackals sampled from India**

| ***Specimen ID*** | ***Haplotype*** | ***Genbank Acc. #*** | ***Sample type*** | ***Latitude (N)*** | ***Longitude (E )*** | ***Locality*** | ***Date*** |
| --- | --- | --- | --- | --- | --- | --- | --- |
| D237 | Cytb_Ind1 | KT343779 | Tissue | 29.4431 | 77.6629 | Muzzaffarnagar, Uttar Pradesh | 01 December 2003 |
| D246 | Cytb_Ind1 | KT343779 | Tissue | 29.7081 | 76.5832 | Kaithal, Haryana | 05 May 2003 |
| D247 | Cytb_Ind1 | KT343779 | Tissue | 29.5952 | 75.9163 | Kaithal, Haryana | 06 May 2003 |
| D255 | Cytb_Ind1 | KT343779 | Tissue | 28.9432 | 77.6396 | Meerut, Uttar Pradesh | 10 September 2004 |
| D269 | Cytb_Ind1 | KT343779 | Hair | 29.0352 | 78.7574 | Moradabad, Uttar Pradesh | 01 October 2005 |
| D270 | Cytb_Ind1 | KT343779 | Hair | 29.0407 | 78.7468 | Moradabad, Uttar Pradesh | 01 November 2005 |
| D467 | Cytb_Ind1 | KT343779 | Tissue | 29.8904 | 77.4555 | Saharanpur, Uttarpradesh | 13 November 2007 |
| D468 | Cytb_Ind1 | KT343779 | Tissue | 29.6338 | 78.3866 | Nazimabad, Uttarakhand | 11 November 2007 |
| D470 | Cytb_Ind1 | KT343779 | Tissue | 29.3859 | 78.6780 | Afzalgarh, Uttar Pradesh | 11 September 2007 |
| D471 | Cytb_Ind1 | KT343779 | Tissue | 26.1878 | 75.8373 | Tonk, Rajasthan | 26 December 2007 |
| D476 | Cytb_Ind1 | KT343779 | Tissue | 25.9779 | 76.3318 | Bakija, Ranthambore Tiger Reserve, Rajasthan | 11 February 2007 |
| D156 | Cytb_Ind2 | KT343780 | Tissue | 22.0583 | 72.0244 | Velavadar National park, Gujarat | 14 November 2000 |
| D41 | Cytb_Ind2 | KT343780 | Tissue | 23.3700 | 69.2393 | Kachchh, Gujarat | 23 January 1997 |
| D43 | Cytb_Ind2 | KT343780 | Blood | 23.2538 | 69.0536 | Kachchh, Gujarat | 24 January 1997 |
| D469 | Cytb_Ind2 | KT343780 | Tissue | 29.3282 | 78.4902 | Dhampur, Uttar Pradesh | 11 November 2007 |
| D473 | Cytb_Ind2 | KT343780 | Tissue | 23.7369 | 80.3275 | Mandla-Jabalpur Highway, Madhya Pradesh | 11 September 2007 |
| D474 | Cytb_Ind2 | KT343780 | Tissue | 23.0776 | 79.6165 | Mandla-Jabalpur Highway, Madhya Pradesh | 12 September 2007 |
| D480 | Cytb_Ind2 | KT343780 | Tissue | 29.0584 | 77.2669 | Badoth, Uttarpradesh | 20 February 2008 |
| D155 | Cytb_Ind3 | KT343781 | Tissue | 22.0642 | 72.0392 | Velavadar National park, Gujarat | 16 November 2000 |
| D172 | Cytb_Ind3 | KT343781 | Blood | 22.0428 | 72.0384 | Velavadar National park, Gujarat | 12 December 2000 |
| D34 | Cytb_Ind3 | KT343781 | Tissue | 22.0413 | 72.0292 | Velavadar National park, Gujarat | 01 January 1995 |
| D36 | Cytb_Ind3 | KT343781 | Tissue | 22.0563 | 72.0708 | Bhal, Gujarat | 17 June 1995 |
| D39 | Cytb_Ind3 | KT343781 | Blood | 22.9485 | 72.1319 | Vegad, Bhal, Gujarat | 22 February 1996 |
| D46 | Cytb_Ind3 | KT343781 | Tissue | 21.9720 | 72.1118 | Bhal, Gujarat | 21 February 1996 |
| D475-i | Cytb_Ind4 | KT343782 | Hair | 12.6715 | 76.6251 | Melkote, Karnataka | 01 April 2012 |
| D475-ii | Cytb_Ind4 | KT343782 | Hair | 12.6715 | 76.6251 | Melkote, Karnataka | 02 April 2012 |
| D29b | Cytb_Ind5 | KT343783 | Tissue | 21.9042 | 71.8784 | Vallabhipur, Bhal, Gujarat | 01 June 1996 |
| D31 | Cytb_Ind5 | KT343783 | Tissue | 23.2956 | 70.0596 | Hirapar, Kachchh, Gujarat | 01 June 1997 |
| D42 | Cytb_Ind5 | KT343791 | Tissue | 21.8739 | 71.8736 | Vallabhipur, Bhal, Gujarat | 06 January 1997 |
| D248 | Cytb_Ind6 | KT343784 | Tissue | 29.8285 | 77.8647 | Roorkee, Uttarakhand | 01 November 2003 |
| D249 | Cytb_Ind6 | KT343784 | Hair | 29.3610 | 77.7949 | Muzzaffarnagar, Uttar Pradesh | 01 December 2003 |
