# Supplementary material for: Phylogeography of the Golden Jackal (Canis aureus) in India
Source: PLoS One. 2015 Sep 28;10(9):e0138497. doi: 10.1371/journal.pone.0138497 (PMC4586146; doi:10.1371/journal.pone.0138497)
Supplement: S3 Table — Estimating the best model of molecular substitution inferred using Log Bayes Factors (LBF) from Bayesian posterior distributions in Mr Bayes. Rate variation for tested models—gamma distributed rate variation across sites (gamma); gamma distributed with proportion of invariable sites (invgamma); rate variation with proportion of invariable sites (propinv); equal rate variation across sites (equal). (DOCX) [file pone.0138497.s005.docx]

| **S3 Table.** **Best model selection.** Estimating the best model of molecular substitution inferred using Log Bayes Factors (LBF) from Bayesian posterior distributions in Mr Bayes. Rate variation for tested models - gamma distributed rate variation across sites (gamma); gamma distributed with proportion of invariable sites (invgamma); rate variation with proportion of invariable sites (propinv); equal rate variation across sites (equal). | | | | |
| --- | --- | --- | --- | --- |
| **Model** | **Harmonic Mean** | **LBF** | **Model**  **probability** |  |
| GTR + invgamma | -3081.46 | 0 | 0.980719 |  |
| GTR + propinv | -3083.54 | -4.154 | 0.015399 |  |
| GTR + gamma | -3099.49 | -36.062 | 2.14E-16 |  |
| GTR + equal | -3201.17 | -239.428 | 1.02E-104 |  |
| F81 + invgamma | -3451.05 | -739.180 | 0.00E+00 |  |
| F81 + propinv | -3409.53 | -656.136 | 1.08E-285 |  |
| F81 + gamma | -3447.53 | -732.140 | 0.00E+00 |  |
| F81 + equal | -3513.69 | -864.460 | 0.00E+00 |  |
| HKY + invgamma | -3126.01 | -89.100 | 1.98E-39 |  |
| HKY + propinv | -3084.23 | -5.532 | 0.003882 |  |
| HKY + gamma | -3093.67 | -24.426 | 2.42E-11 |  |
| HKY + equal | -3207.02 | -251.114 | 8.59E-110 |  |
| mixed + invgamma | -3099.09 | -35.256 | 4.79E-16 |  |
| mixed + propinv | -3088.50 | -14.084 | 7.50E-07 |  |
| mixed + gamma | -3091.90 | -20.884 | 8.35E-10 |  |
| mixed + equal | -3207.86 | -252.802 | 1.59E-110 |  |
